# Tag Localization with Asynchronous Inertial-Based Shifting and Trilateration

**DOI:** 10.3390/s19235204

**Published:** 2019-11-27

**Authors:** Abdallah Y. Alma’aitah, Lobna M. Eslim, Hossam S. Hassanein

**Affiliations:** 1 Department of Network Engineering and Security, Jordan University of Science and Technology, Irbid 22110, Jordan; ayalmaaitah@just.edu.jo; 2 School of Computing, Queen’s University, Kingston, ON K7L 3N6, Canada; lobna.eslim@cs.queensu.ca

**Keywords:** inertial sensor, localization, RSSI, crowdsourcing

## Abstract

Personal Area Networks (PAN) are key topologies in pervasive Internet of Things (IoT) localization applications. In the numerous object localization techniques, centralization and synchronization between the elements are assumed. In this paper, we leverage crowdsourcing from multiple fixed and mobile elements to enhance object localization. A cooperative crowdsourcing scheme is proposed to localize mobile low power tags using distributed and mobile/fixed readers for GPS assisted environments (i.e., outdoor) and fixed readers for indoors. We propose Inertial-Based Shifting and Trilateration (IBST) technique to provide an accurate reckoning of the absolute location of mobile tags. The novelty in our technique is its capability to estimate tag locations even when the tag is not covered by three readers to perform trilateration. In addition, IBST provides scalability since no processing is required by the low power tags. IBST technique is validated through extensive simulations using MATLAB. Simulation results show that IBST consistently estimates location, while other indoor localization solutions fail to provide such estimates as the state-of-the-art techniques require localization data to be available simultaneously to provide location estimation.

## 1. Introduction

Radio Frequency Identification (RFID) and IEEE 802.15-based tags are widely accepted to be the de facto of low power identification and computation in wireless communication. These tags, battery assisted ones in particular, can be equipped with sensing elements and memories to provide rich information for services requiring location estimation. Therefore, Personal Area Network (PAN) technologies are considered to be a key enabler of many Internet of Things (IoT) smart applications. Smart applications rely on contextual information, predominantly the location of the tags or “things” at a specific time [1]. Location awareness of the tags will aid contextual decision-making, hence enhancing the quality of experience for users. Nevertheless, localizing the tags, while considering the IoT characteristics in terms of scalability, heterogeneity, and mobility, remains a challenging problem.

During the last decade, PAN technology has developed rapidly in conjunction with a steep cost reduction of the supporting system-on-chip integrated circuits [2]. Due to this development, several low-power, small footprint, and sensor-assisted devices have sprouted. Consequently, myriad of pervasive and context-aware applications have been proposed [3,4,5,6,7,8,9,10,11]. In particular, in RFID systems, tag- and reader-based localization schemes were proposed. In tag-based localization, the tag location is mainly estimated based on its proximity to a given reader(s) with a known location [12]. In reader-based localization, reader location can be estimated similarly by its proximity to a pre-deployed set of tags [5]. Unsurprisingly, as readers’ prices are orders of magnitude more than tags, RFID tag-localization is the dominant in localization solutions. 

In this paper, we propose a cooperative tag-localization scheme called Inertial-Based Shifting and Trilateration (IBST) based on crowdsourcing in both indoor and outdoor environments. The system is comprised of battery-assisted tags that are attached to mobile objects, while mobile or fixed readers (with known absolute locations) will read and write corresponding information on these tags. In our scheme, the *Readers* (heterogeneous, independent, and dynamic PAN-based standardized readers) detect surrounding tags, read their current memory, and update the tags’ memory with the estimated distance and current reader location information. The readers report the detected tags’ information to a backend server, the server processes the data from all readers, and then it responds to users’ queries about objects of interest. We remark that our approach is fundamentally different from existing tag localization techniques.

Existing localization techniques assume that the tag’s location can be estimated based on simultaneous information from all surrounding readers. This assumption, however, may not always be applicable in mobile or dynamic environments. For instance, at a given time, if the current information for localization is obtained from less than three readers, location cannot be reckoned [13]. This challenge is evident as the non-uniformity of readers’ locations, heterogeneity in readers’ detection ranges, and mobility of both tags and readers prevent having sufficient and synchronous detection information for each tag. 

The proposed asynchronous crowdsourcing localization technique, Inertial-Based Shifting and Trilateration (IBST), is the focus of this paper. Whenever sufficient detection information is not available, IBST utilizes asynchronous reader detections to localize tags that are equipped with inertial sensors. The tags are localized based on Received Signal Strength Indicator (RSSI) trilateration/proximity and inertial-based shifting. To the best of our knowledge, our approach is the first to develop a PAN object localization system utilizing reader crowdsourcing,

- utilize tags’ memory to store reader detection information and location information that can be read by other passing readers, and
- use asynchronous detection information and internal inertial sensor information to enhance localization when the concurrent spatial information is not sufficient to localize a tag.

We validate the proposed system through extensive simulations using MATLAB. Results show that our approach can achieve accurate location estimation in typical IoT settings.

The remainder of this paper is organized as follows: Section 2 reviews some of the related work and motivates our proposed approach. In Section 3, we provide system models, such as system architecture, channels model, and inertial sensors displacement model. The proposed IBST crowdsourcing technique is described in Section 4. Section 5 presents the performance evaluation of the proposed system. Finally, our conclusion is given in Section 6.

## 2. Related Work and Motivation

Various PAN localization schemes have been proposed in the literature, in which, an infrastructure of RF transceivers (e.g., RFID readers, Zigbee sink nodes, etc.) are deployed to detect and collect information from surrounding tags [6,7,8,9,10]. In [6], the Coupled RSSI and Inertial Navigation System (INS) Localization scheme (CRIL) has been proposed, having two main advantages. CRIL can adapt effectively and quickly to dynamic communication environments, and it can account for the uncertainties in RSSI measurements, among which are varying covariance and outliers. The indoor localization approach, named iBILL [7], uses iBeacon and inertial sensors simultaneously in large open areas. Users’ real-time locations are estimated by inertial sensors through an improved particle filter. The latter is used to alleviate the effect of sensing fluctuations of localization errors. In HILS [8], a more efficient Heron-bilateration-based position determination technique (HBPD) is proposed. This novel technique requires Wi-Fi signals from only two access points to localize a mobile device. HILS utilizes HBPD technique to reset the bias drift errors associated with inertial sensors and to indemnify the unavailability of strong Wi-Fi signals from Access Points (APs). 

ANTspin [9] has been proposed as an efficient absolute localization method for RFID tags using Spinning Antenna. It introduces a rotary table in the experiment where the reader antennas are fixed to continuously collect dynamics data. Based on the characteristics of collected RSSI data, the relative incident angle and distance between tags and antenna are analyzed for localization. An approach for RSSI-based, calibration-free, and real-time indoor localization is proposed in [10]. Although the author presents switch-beam array-based hardware, compliant with IEEE 802.15.4 router functionality, the focus is on the creation of an algorithm layer to be used with the pre-existing hardware that is capable to enable full localization and data contextualization over a standard 802.15.4 Wireless Sensor Network using RSSI information with no need for a prolonged offline calibration phase.

In each of these five solutions, having sufficient information to localize at a given time is required. In addition, the centralized and fixed infrastructure-based systems provide limited scalability, especially in solutions that require bulky readers that cannot be mobile and may not be applicable for IoT settings, especially in outdoor environments. In the next section, we show the advantage of crowdsourcing on the adaptability of the system given a dynamic environment.

## 3. System Model

### 3.1. System Architecture 

We consider an (indoor or outdoor) environment where we track tagged mobile users (a person, animal, or object) in two-dimensional space in a specific place. A number of dynamic, heterogeneous, and uncoordinated readers in known positions are distributed and authorized to discover, access, and update the memories of tags within their vicinity. The tags are assumed to be battery-assisted and can be read through a PAN standardized protocol (e.g., IEEE 802.15.4 [14], RFID [15], WiFi [16], etc.). Tags are equipped with an ultra-low power inertial measurement unit (IMU) [17]. Thus, the object within the range of a given reader can communicate data and measure the received signal strength. 

### 3.2. Ranging Model

In our proposal, we adopt RSSI-based ranging in which the distance is estimated based on the received signal strength due to its simplicity and availability in most wireless transceivers. However, other ranging techniques (e.g., Angle of Arrival and Time difference of Arrival) may be incorporated (especially in fixed dedicated readers) if higher hardware complexity can be tolerated. 

Based on the lognormal path loss model [18], the RSSI of the transmitted signal from the tag, denoted by *P_RX_*, is written as

(1) $$P_{RX}\left(dBm\right)=A-10\eta \log_{10}\left(\frac{d}{d_{0}}\right)+N_{0}\;,$$

where *A* is the received signal strength at a reference distance $d_{0}$, d is the distance between the reader and the tag, η is the path loss exponent, and $N_{0}$ is the noise in the environment. The value of A depends on the transmitted signal power $P_{TX}$ and the antenna gains of the transmitter and the receiver. The noise $N_{0}$ is usually defined as a zero-mean Gaussian random variable N(0, σ). It is worth mentioning that the antenna in the proposed system is assumed to be omnidirectional. 

Rewriting Equation (1), the distance d between the reader and the tag can be written as:

(2) $$d=d_{0}\cdot 10^{\frac{A-P_{RX}+N_{0}}{10\eta }}\;.$$

Shadowing and multipath effects are severe on RSSI ranging at long distances due to noise factor in the power part of Equation (2). In other words, it is more accurate to estimate the distance if the power of the received signal is higher than estimating the distance at lower RSSI. 

### 3.3. Inertial Sensor Model

In our scheme, we utilize inertial sensor modules in the tags to improve the localization accuracy. In the Inertial Navigation System (INS) [17], IMU is used to estimate a tag’s location from a given reference point. The sensor is used to estimate the orientation (heading) from that reference point. However, the location error of the IMU usually accumulates, leaving the sole use of inertial sensors impractical. 

**Figure 1:**
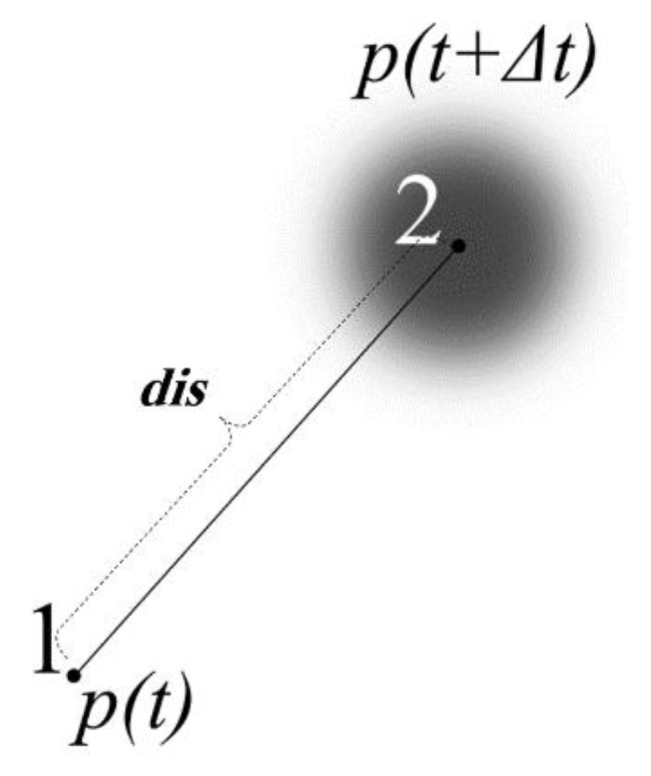
Illustration of the inertial tag displacement model in Equation (3).

To model the INS sensor estimated location, accumulated error per displacement within a time period Δt is presented based on errors in the sensor which output the degrees, the gyroscope which outputs the angular velocity of the object, and the accelerometer which outputs the linear acceleration. Accordingly, if the current two-dimensional location is $P_{i}$ at time *t*, the next estimated location, $P_{i+1},$ at t+Δt can be given as

(3) $$P\left(t+\Delta t\right)=P\left(t\right)\;+\;dis\;+\;dis_{e},$$

where *dis* is the mean displacement vector calculated for Δt, and $dis_{e}$ is the displacement error, including sensor biases, which is a function of angular velocity and linear acceleration errors. Figure 1 illustrates the inertial tag displacement model.

As the inertial error reduction is beyond the scope of this paper, the error is numerically modeled based on the results reported in [19]. 

## 4. Proposed Solution

### 4.1. Crowdsourcing Scheme 

Our approach suggests that in dynamic environments, the available crowdsourcing in terms of mobile and fixed readers, along with tags’ memories, can be leveraged to provide localization service. 

Given a set of *N* tags and a set of *M* readers, when a *reader* $R_{m}\;\left(m\in \left\{1,2,\ldots \;M\right\}\right)\;$ detects a tag $T_{n}\;\left(n\in \left\{1,2,\ldots \;N\right\}\right)$ successfully, it generates a detection event containing time and range information about the tag with respect to the *reader*. Each reader adds the event to a Detections table in the tag’s memory, and these events are subsequently used to localize the tag. 

At any tag detection, the reader creates and then updates two types of information:

- *Detections table*, shown in Table 1, contains temporal and spatial information about a tag $T_{n}$ with respect to reader $R_{m}$. Such information is considered the raw information about a tag’s vicinity to a specific reader at a given time. The number of entries in Detections table is denoted by *k*.
- *Absolute Tag Location* (ATL) table, shown in Table 2, contains the estimated locations of a tag $T_{n}$. Each location is identified by its estimation time.
- *Tag Displacement Vector* (TDV) table contains the distance vectors that are measured based on inertial sensors (IS) records. TDV contains *k* − 1 tag displacement vectors between every two subsequent reader detections.

*Readers* scan the surrounding vicinity to detect tags in their proximity, fetch *Detections*, ATL, and TDV tables, in addition to IS records. If enough detection entries are available to perform trilateration, the tags’ location is estimated, and tables in the tag’s memory are updated. 

By this schema, tags’ memories are always updated once detected by *Readers*. For each successfully identified tag *T_n_* by a given reader *R_m_*, the reader creates a detection record in the memory of *T_n_*.

The tables in the tag’s memory allow the reader to estimate the tag’s current location. Note that the location estimation algorithm is performed by readers. Tags function is limited to inertial sensors information recording. Location information processed by the reader is then reported to a central database (online or offline), which is accessible by a user that is interested in the location of a specific tag(s). 

### 4.2. Inertial-Based Shifting Trilateration (IBST) Technique

Typically, most distance-based localization techniques assume that the measured spatial information, even those from mobile anchors, is synchronous and sufficient to localize objects. Thus, they estimate the object position based on the intersection of the given spatial information (i.e., trilateration, bounding box, etc.). In dynamic reader deployment environments, this assumption may not be applicable, which results in blind spots where no location estimation is possible. In fixed and pre-deployed reader environments, high-density reader deployment will be needed to ensure full coverage of the targeted area. 

Inertial-Based Shifting Trilateration (IBST) is proposed to overcome insufficient spatial information (i.e., non-intersecting ranges from three or more readers is available). In IBST, once new ranging measurements by RSSI are available (i.e., once a tag is detected by a reader), we expand the range around the previously calculated location(s) based on the tag’s IS records. Next, we introduce IBST shifting process and algorithm.

### 4.3. IBST Process

In IBST, the range from the reader to a detected tag is considered a circle that is centered at the *Reader* position. The radius of such a circle is the mapped distance from the RSSI channel model and is bounded by the maximum reading range of the reader.

IBST makes the previously estimated location(s) centers for circles with radii based on the recorded inertial sensor readings upon current detection. Ultimately, to apply trilateration, at least two previous estimated locations in addition to the current detection range by the reader are needed.

**Definition** **1.** *(detection set): Given a set of K Readers, the detection set of a tag i is the spatial information measured simultaneously or consecutively, is denoted by* $l_{k}^{i}$ *, where k* ∈ *{K}, and is ordered chronologically in the tag’s memory.*

Each element $l_{k}^{i}$ in the *Detections* table of tag i is represented by {*t_k_*, *R_k_*(*x*,*y*), *d_k_*}, which are defined in Table 1 as detection time, reader position, and distance (range). The tag can be localized once it has three entries in its *Detections* table.

**Theorem** **1.** *If a given tag *i*with a known initial location*$L_{i}$*with a relative two-dimensional coordinates*$(x_{L_{i}}$, $y_{L_{i}})$*has been detected at a range of*$d_{1}$*from a reader*$\;R_{1}$*of a known two-dimensional coordinates*$(x_{1}$, $y_{1})$*, then at most, two points (*$P_{1}$*and*$P_{2}$*) of the tag’s absolute location can be determined.*

**Figure 2:**
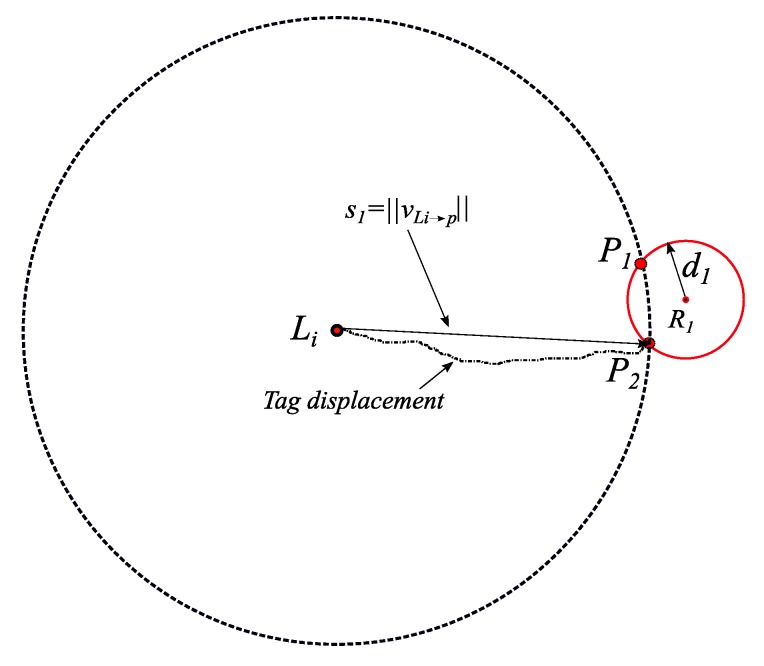
Illustration of the proof of Theorem 1 for three different displacement vectors from reader 1 to reader 2.

**Proof.**  As long as the inertial sensor displacement vector $v_{L_{i}\to p}$, from the point $L_{i}$ to the moment of being detected by $R_{1}$, is available, the circle centered at $(x_{L_{i}}$, $\;y_{L_{i}})$ with a radius of $s_{1}=\|v_{L_{i}\to p}\|$ and the circle centered at $(x_{1}$, $\;y_{1})$ with a radius of $d_{1}=\|v_{R_{1}\to p}\|$ will intersect. If $v_{L_{i}\to p}$ is perpendicular on the circle around $R_{1}$, one intersecting point will result; otherwise, two intersecting points will result as $\|v_{L_{i}\to p}\|+$ $\left\|v_{R_{1}\to p}\|>\|v_{L_{i}\to R_{1}}\right\|$. An illustration is shown in Figure 2. □

**Theorem** **2.** *If a given tag*i*of a known initial location*$L_{i}$*with a relative two-dimensional coordinates*$\;(x_{L_{i}}$, $\;y_{L_{i}})$*has been sequentially or simultaneously detected at ranges of*$d_{1}$*and*$d_{2}$*from readers*$\;R_{1}$*and*$R_{2}$*, respectively (the relative two-dimensional coordinates of the readers are*$(x_{1}$, $\;y_{1})\;$*and*$(x_{2}$, $\;y_{2})$*), then, one solution of the tag’s absolute location can be determined.*

**Figure 3:**
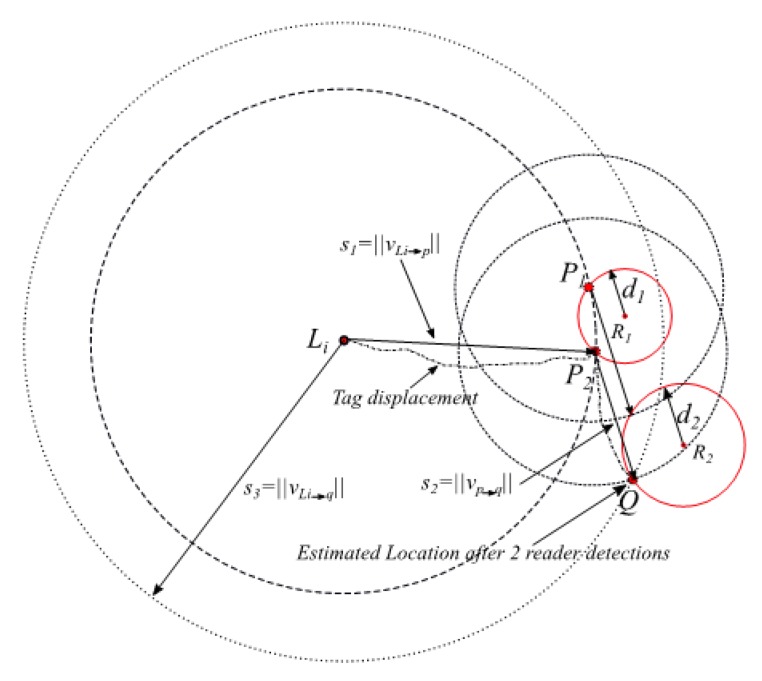
Illustration of the proof of Theorem 2 for a tag being detected by two readers. The shifted readers 1 and 2 in dotted gray circles are non-collinear, which results in a single location solution.

**Proof.**  As long as the inertial sensor displacement vectors $v_{L_{i}\to p}$ and $v_{p\to q}$ are available, trilateration of the circle $C_{1}$ centered at $(x_{L_{i}}$, $y_{L_{i}})$ with a radius of $s_{3}=\|v_{L_{i}\to p}+v_{p\to q}\|$, the circle $C_{2}\;$ centered at $(x_{1}$, $y_{1})$ with a radius of $s_{2}=\|v_{p\to q}\|$, and the circle $C_{3}$ centered at $(x_{2}$, $y_{2})$ with a radius of $d_{2}=\|v_{R_{2}\to q}\|$ will intersect in a single point Q, which is a point on all three circles if the intersection points between $C_{1}$ and $C_{2}$ are not symmetrical around the vector passing through the initial location $(x_{L_{i}}$, $y_{L_{i}})$ and current reader $(x_{2}$, $y_{2})$. An illustration is shown in Figure 3. □

**Figure 4:**
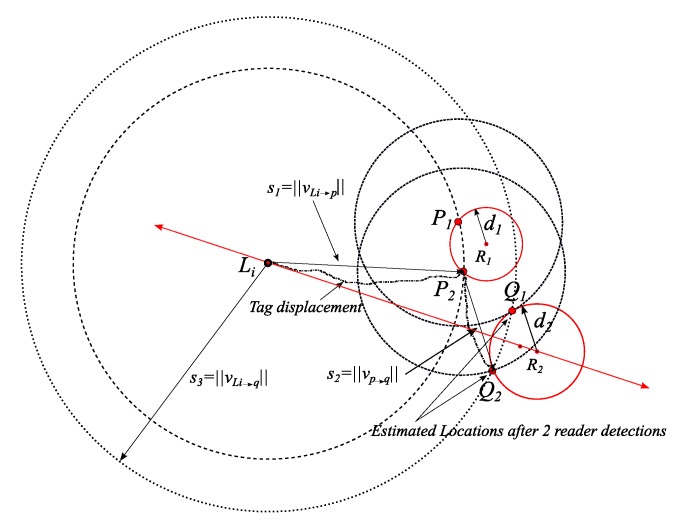
Illustration of the case of collinearity between the shifted readers 1 and 2 in dotted gray circles and the third reader. This case results in two location solutions.

It is worth mentioning that in a case of collinearity in the shifted readers’ centers, a unique solution of the tag’s location may not be calculated and further detection(s) would be required. Figure 4 illustrates the collinearity effect on localization after two detections from the initial location.

### 4.4. IBST Algorithm with Ideal Inertial-Magnetic and Range Readings

IBST is executed, as in Algorithm I, to maintain location estimation in fixed and dynamic reader settings. The input is a number of asynchronous detections and the output is the updated Absolute Tag Location (ATL) tables. In Algorithm I, inertial sensors readings are stored in the tags’ memories between subsequent detections from readers (lines 2–4). Once the tag is detected by a reader, the reader reads the Inertial Sensors (IS) records and updates the Detections table with the time of detection, reader’s location, and range between the tag and the reader (lines 5–6). If the tag has been detected previously by another reader, the current reader calculates the displacement vector from the previous reader to the current one (lines 7–9). 

Once the tag with a known initial location has three entries in the Detections table, trilateration equations are applied to calculate the tag’s current location. The current location is the intersection of three circles: the two circles around latest two Absolute Tag Location (ATL) entries (with radii that are based on the shifts from inertial sensor vectors) and the circle around the current reader (with estimated range as the radius). Once the location is calculated, the oldest detection entry has no bearing since next detection will be the new third detection (line 15). If the trilateration results in one location (unique solution), the readings are not collinear, so the location is reported to central database, and the ATL table in the tag is updated (lines 16–18). Otherwise, the three circles intersect in two points (i.e., no unique solution), and further reading(s) would be needed to calculate a unique solution (lines 19–20). If two entries are in the Detections table (including the current detection), the reader calculates the intersection(s) between the circles around latest ATL entry (with radius that is based on the shifts from inertial sensor vectors) and the circle around the current reader (lines 22–25). If less than three entries are in the Detections table, including the current reader, the vector between the current detection and the previous one is stored in the Tag Displacement Vector (TDV) table, and further detection(s) are required to perform trilateration (lines 26–27).
**Algorithm I.** Ideal Inertial-Based Shifting Trilateration.Input: Asynchronous readers’ detections, raw inertial sensor dataOutput: Updated Absolute Tag Location (ATL)1: initialize tag memory: empty Detections, IS, TDV, and ATL tables2: while (tag is not detected)3:   Do   record data from inertial sensors in IS table4: End While5: Read Detections and IS tables // *a tag is detected*6: Update Detections table with $l_{k}^{n}$7: If (Detections table has k > 0 entries)8:  Calculate displacement vector(s) $v_{\left(k-1\right)\to k}$ based on IS table9: End If10: If (Detections table has k = 2)  // *check for enough detections to perform trilateration*11:  $C_{0}$ = Circle around $ATL_{0}$ coordinates in $l_{0}^{n}$ by a radius of $\left\|v_{0\to 1}+v_{1\to 2}\right\|$12:  $C_{1}$ = Circle around $ATL_{1}$ coordinates in $l_{1}^{n}$ by a radius of $\left\|v_{1\to 2}\right\|$13:  $C_{2}$ = the circle around the current reader by a radius of RSSI mapped detection range 14:  Calculate the current absolute tag location by trilateration of $C_{0},C_{1},$ and $C_{2}$15:  Delete $l_{0}^{n}$ and $v_{0\to 1}$; // *delete first entry in Detections and TDV tables*16:  If (solution is unique)17:   Report current ATL (ATL_i_) to a central database server18:   Update ATL table; go to 2  // *add the new ATL to previous ATL entries*19:  Else20:   go to 221:  End If22: Else (Detections table has k = 1)23:   $C_{1}$ = Circle around $L_{2}$ coordinates in $l_{2}^{n}$ by a radius of $\left\|v_{1\to 2}\right\|$24:   $C_{2}$ = the circle around the current reader by a radius of RSSI mapped detection range 25:   Calculate the current absolute tag location(s) by intersecting $C_{1}$ and $C_{2}$26: Else27:  Update Detections table with $l_{k}^{n}$ and TDV table with $v_{\left(k-1\right)\to k}$, go to 228: End If


Note that Algorithm I shows how the IBST works under the assumptions of perfect estimation of range and inertial readings. Algorithm I (ideal IBST) is modified to Algorithm II (practical IBST) to account for errors in inertial sensors, in addition to RSSI ranging errors.

### 4.5. IBST Algorithm with Incorporated Errors in Inertial and Range Readings

As mentioned in Section 3, the drift in inertial sensor readings will result in a margin of error which renders the estimated location useless. RSSI ranging, including its error margin, will provide a continuous reckoning of the estimated absolute location and will harness inertial sensor drifting error. Algorithm II below is a modified version of Algorithm I to accommodate such drifts in IS readings and RSSI ranging margins. 

Similar to Algorithm I, once a tag is detected, its Detections table is updated with time, reader’s location, and two ranges of information, one based on mean RSSI and the other on RSSI and one standard deviation in noise (*σ_r_*) (see line 6). 

**Figure 5:**
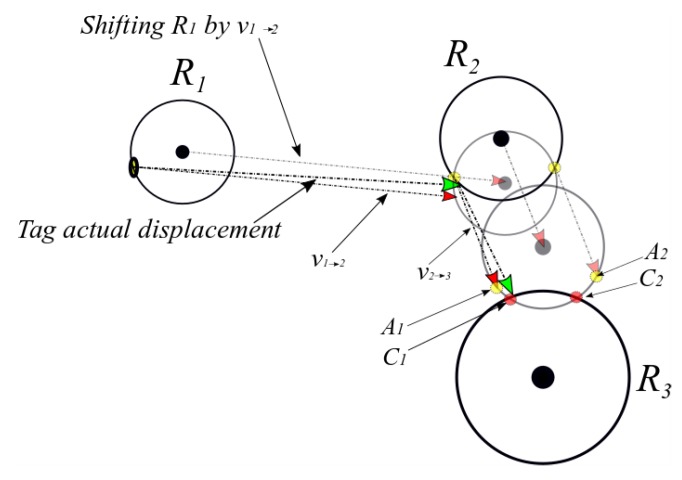
Example of the intersection points set (C) between shifted $R_{2}$ ($R_{2}^{\to }$) and $R_{3}$ in red circles. Example of shifted previous intersection points set (A) in yellow circles.

In Algorithm I, the tag location calculation was based on ideal IS readings (i.e., exactly as the actual tag trajectory); hence, trilateration will result in one unique solution as shown in Figure 3 (recall that collinearity will result in two solutions.). With drifting errors in IS, trilateration may result in two solutions (even when readers are not collinear) or no solution at all. Therefore, once a tag is detected by the first reader, the first reader calculates the intersection between the two circles with centers of $L_{i}$ and $R_{1}$ and radii of $s_{1}$ and $d_{1}$, respectively. The resulting intersection points are at most two and both are considered possible ATLs and denoted as $ATL_{1,1}$ and $ATL_{1,2}$, as illustrated in Figure 5. After the first reader detection and updating the ATL table with all possible ATLs, the tag records the inertial sensor information until the next reader detection.

Once the tag is detected by the second reader at an estimated distance of $d_{2}$ from its center coordinates, the reader fetches previous entries in the Detections table, ATL table, and IS table. Based on the entries in the three tables, the reader defines three sets of circles:

1. Circle $C_{0}$ with its center at the second latest ATL(s) and radius of $s_{0}=\|v_{1\to 2}+v_{2\to 3}\|$,
2. Circle $C_{1}$ with its center at latest (i.e., most recent) ATL(s) and radius of $s_{1}=\|v_{2\to 3}\|$, and
3. Circle $C_{2}$ with its center at the reader $R_{2}$ and radius of $d_{2}$.

Then $R_{2}$ calculates the intersection points between circles $C_{1}$ and $C_{0}$. As shown in Figure 5, the latest ATLs are $ATL_{1,1}$ and $ATL_{1,2}$, and the second, latest ATL is $ATL_{0}=L_{i}$. Each circle in $C_{1}$ may intersect with $C_{0}$ at most in two points. For instance, the circle $C_{1}$ around $ATL_{1,1}$ intersects with the circle $C_{0}$ around $ATL_{0}$ in two points denoted by the points $A_{1}$ and $A_{2}$. Similarly, $C_{1}$ around $ATL_{1,2}$ and will intersect with $C_{0}$ around $ATL_{0}$ at the points $A_{3}$ and $A_{4}$. As will be described shortly, these intersection points (i.e., $A_{1}$ … $A_{4}$) will be the means for selecting the next ATL(s).

**Figure 6:**
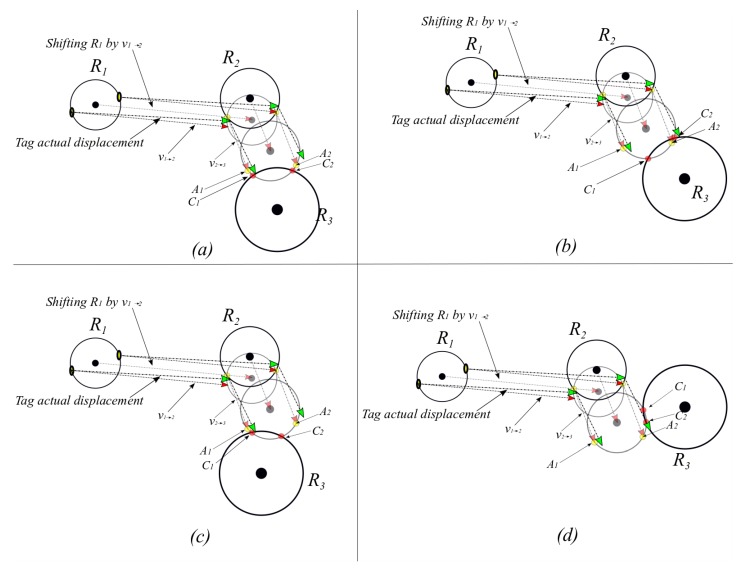
The four possible scenarios for the selection between points $C_{1}$ and $C_{2}$ addressed in lines 15–23 of Algorithm II.

**Figure 7:**
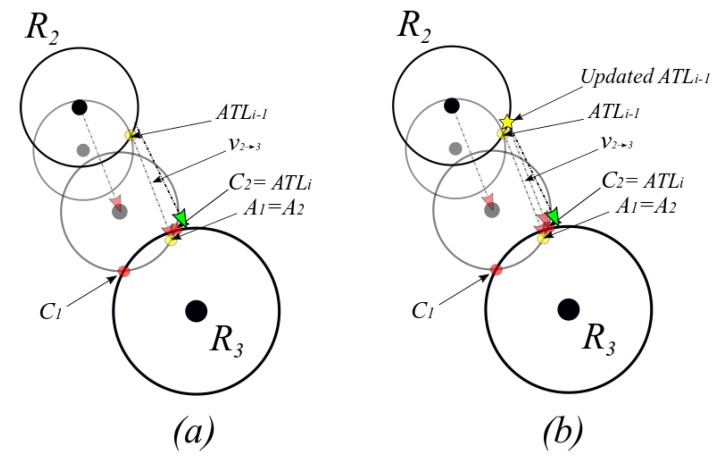
Example of the updating process of $ATL_{i-1}$. (**a**) Selection of feasible point in C (**b**) based on the selected C ($C_{2}$ in this example = $ATL_{i}$); $ATL_{i-1}$ is updated by $ATL_{i}$- $v_{2\to 3}$ denoted by the yellow star.

$R_{2}$ also calculates the intersection points between $C_{1}$ and $C_{2}$. The intersection points (noted as B points) are the candidates for the current ATL (i.e., $ATL_{2}$). In case of two points in the latest ATL (as in the example in Figure 5 where latest ATL has $ATL_{1,1}$ and $L_{1,2}$), three possible cases may occur:

1. $C_{2}$ intersects with one of the $C_{1}$ circles (e.g., $C_{1\_2}$ as shown in Figure 5). In this case, $\left\|A_{i}-B_{j}\right\|$ will be calculated for all A’s on $C_{1\_2}$ and B’s on $C_{2}$. Then, B with $\min\left(\left\|A_{i}-B_{j}\right\|\right)$ will be considered $ATL_{2}$.
2. $C_{2}$ intersects with two $C_{1}$ circles (e.g., $C_{1\_1}$ and $C_{1\_2}$ circles shown in Figure 6). In this case, $\left\|A_{i}-B_{j}\right\|$ will be calculated for all A’s on $C_{1\_1}$ and B’s on $C_{2}$. Then B with $\min\left(\left\|A_{i}-B_{j}\right\|\right)$ will be considered $ATL_{2\_1}$. Similarly, $\left\|A_{i}-B_{j}\right\|$ will be calculated for all A’s on $C_{1\_2}$ and B’s on $C_{2}$. Then, B with $\min\left(\left\|A_{i}-B_{j}\right\|\right)$ will be considered $ATL_{2\_2}$. Since two points result from the intersection of such a case, $ATL_{2\_1}$ and $ATL_{2\_2}$ replace $ATL_{1,1}$ and $ATL_{1,2}$.
3. $C_{2}$ does not intersect with any of $C_{1}$ circles as shown in Figure 7. $ATL_{2}$ is considered the intersection point between $C_{2}$ and the line which connects A with the minimum distance to the reader $R_{2}$.

**Figure 8:**
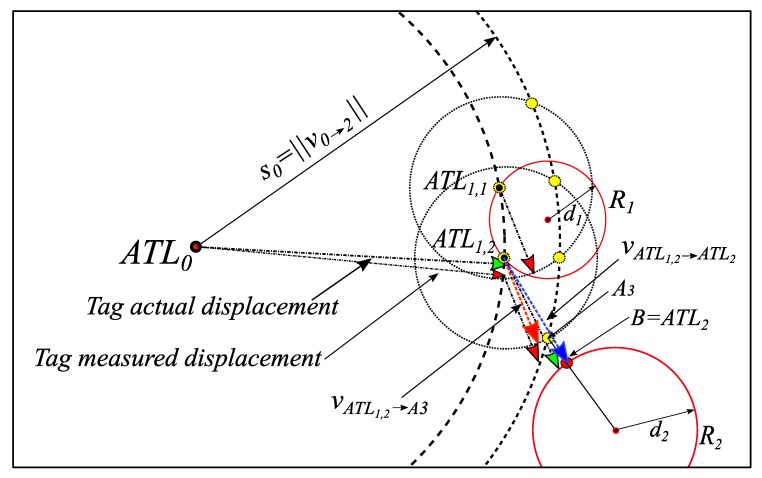
Example of the updating process of displacement vector when $C_{2}$ and $C_{1}$ do not intersect. The updated vector is $v_{ATL_{1,2}\to \;ATL_{2}}$ (in blue), and inertial sensor reading vector is $v_{ATL_{1,2}\to \;A_{3}}$ (in red).

In the case of no intersection between $C_{2}$ and $C_{1}$ (third case shown in Figure 7), the resultant ATL will be at a longer displacement from previous ATL than $v_{ATL_{i-1}\to \;A}$. Therefore, the displacement vector in the tag is updated by $\;v_{ATL_{i-1}\to \;ATL_{i}}$. An example of the displacement vector update is depicted in Figure 8. The update will ensure that the displacement vector is on the borders of the current detecting reader $R_{2}$ and the precious reader $R_{1}$.
**Algorithm II.** Inertial-Based Shifting Trilateration.Input: Asynchronous readers’ detections, raw inertial sensor dataOutput: Updated Absolute Tag Location (ATL)1: initialize tag memory: empty Detections, IS, TDV2: while (tag is not detected)3:   Do   record data from inertial sensors in IS table4: End While5: Read Detections, IS, and TDV tables *// a tag is detected*6: Update Detections table with current detection7: If (Detections table has 2 entries)8:  Calculate displacement vector(s) $v_{0\to 1}$ based on IS table9:  $C_{0}$ = Circle around $ATL_{0}$ coordinates in $l_{0}^{n}$ by a radius of $\left\|v_{0\to 1}\right\|$10:  $C_{1}$ = the circle around the current reader by a radius of RSSI mapped detection range11:  Calculate the set of intersection points between $C_{0}$ and $C_{1}$12:  All intersection points I are reported to a central server as $ATL_{1\_i},$
i∈I13: Else if (Detections table has 3 entries)  // *check for enough detections to perform IBST*14:  Calculate displacement vector(s) $v_{1\to 2}$ based on IS table15:  $C_{0}$ = Circle around $ATL_{0}$ coordinates in $l_{0}^{n}$ by a radius of $\left\|v_{0\to 2}\right\|$16:  $C_{1}$ = Circle around $ATL_{1}$ coordinates in $l_{1}^{n}$ by a radius of $\left\|v_{1\to 2}\right\|$17:  $C_{2}$ = the circle around the current reader by a radius of RSSI mapped detection range 18:  Calculate the set of intersection points A’s between $C_{0}$ and $C_{1}$19:  Calculate the set of intersection points B’s between $C_{1}$ and $C_{2}$20:   If (there are 2 intersections between $C_{1}$ and $C_{2}$)21:    $ATL_{2}$ = $B_{.}$ of min $\left(\left\|A_{1}-B_{1}\|,\|A_{1}-B_{2}\|,\|A_{2}-B_{1}\|\|A_{2}-B_{2}\right\|\right)$,22:   Else if (there are 4 intersections between $C_{1}$ and $C_{2}$)23:    $ATL_{2\_1}$ = $B_{.}$ of min $\left(\left\|A_{1}-B_{1}\|\|A_{1}-B_{2}\|\|A_{2}-B_{1}\|\|A_{2}-B_{2}\right\|\right)$,24:    $ATL_{2\_2}$ = $B_{.}$ of min $\left(\left\|A_{3}-B_{3}\|\|A_{3}-B_{4}\|\|A_{4}-B_{3}\|\|A_{4}-B_{4}\right\|\right)$,25:    Update Detections table with $l_{2}^{n}$ and TDV table with $v_{1\to 2}$, go to 226:   Else (there is no intersection between $C_{1}$ and $C_{2}$)27:    Find vector from ${\vec{D}}_{i}=A_{i}\to R_{2},$ find $D_{i}=\|A_{i}-R_{2}\|$ for i=1 to 428:    $ATL_{2}$ = $R_{2}+d_{2}.{\vec{D}}_{i}$ of min $D_{i}$,29:   End if 30:  Report ATLi to a central database server31:  Update Detections table with $l_{3}^{n}$ and TDV table with $v_{1\to 2}$, go to 232: Else 33:  go to 2
34: End If


**Figure 9:**
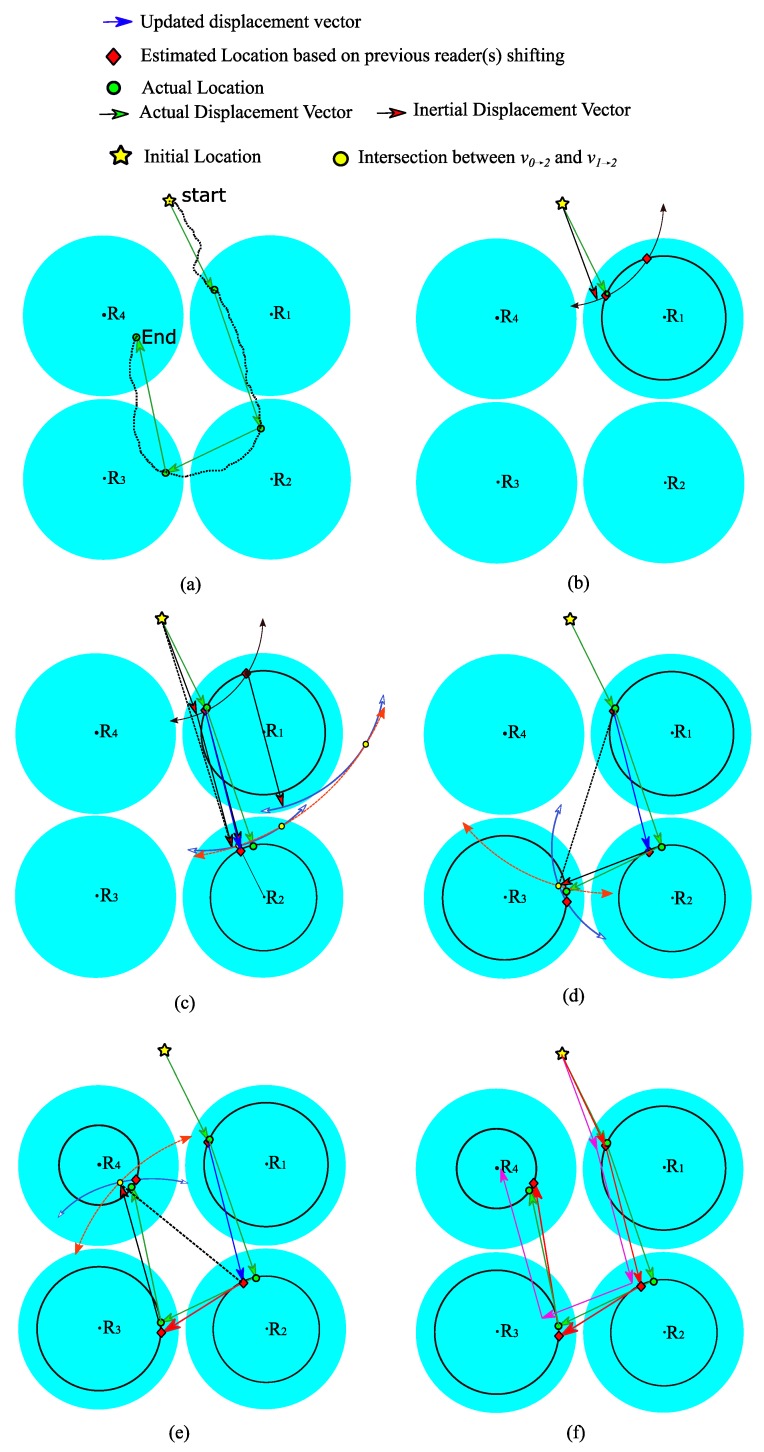
Inertial-Based Shifting and Trilateration (IBST) example of a tag that moves in a path which intersects the four readers $R_{1}$ to $R_{4}$.

In Figure 9, Algorithm II is applied to a scenario in which traditional trilateration is not feasible since there is no intersection between any three readers. The tag track is set to pass through the four readers $R_{1}$ to $R_{4}$, as in Figure 9a. Note that depending solely on ranging or inertial sensors will yield no answer (in RSSI ranging) or high error margins from actual tag track (in inertial sensors readings). In Figure 9b, it is assumed that the tag has a known initial location (i.e., $ATL_{0}$) and moves from the initial location to $R_{1}$, where the intersection points $ATL_{1,1}$ and $ATL_{1,2}$ (the red diamond symbol) are selected based on lines 7–12 in Algorithm II. When the tag is detected by $R_{2}$ as shown in Figure 9c, three entries will be in the tag Detections table and no intersection between circles $C_{2}$ and $C_{1}$ (lines 26–28 in Algorithm II), resulting in $ATL_{2}$ and $ATL_{1,1}$ being replaced by $ATL_{1}$. In Figure 9d,e, there is an intersection between circles $C_{2}$ and $C_{1}$, and lines 13–21 of Algorithm II are executed. The actual track and the resultant track by IBST are marked, respectively, by green and red in Figure 9f; and for comparison, Figure 9f shows the drift in inertial sensor readings (in purple) after four detections.

## 5. Performance Evaluation

In this section, we analyze the performance of our proposed IBST system through extensive simulations and validate the proposed scheme by comparing it with the following two previous methods: (1) RSSI localization system, which estimates the object’s position based on the trilateration of RSSI values only [20]; and (2) INS system, which the results from the inertial and magnetic sensors are used to determine tag location [19]. As will be shown, our proposed system gives precise and stable localization results, even in sparse and random reader deployment scenarios.

### 5.1. Simulation Environment and Parameter Setting

In our numerical evaluation, we consider N mobile readers in an area of 100m by 100m. The tagged object moves in two different paths within this track area. Readers are assumed to have a maximum detection range of 20m. The simulation is executed under sparse and dense deployments of random readers, with 5 and 20 readers, respectively. In addition, two paths are tested as tracks for the tag a rectangular back-and-forth track and a circular track. 

**Figure 10:**
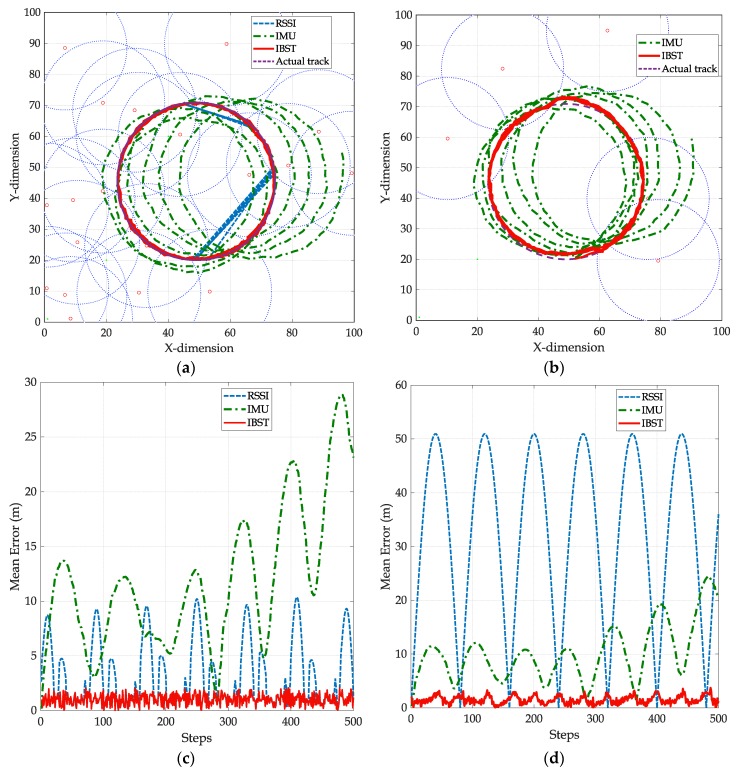
Circular track scenario with dense and sparse and random reader deployment (**a**) The resulting inertial measurement unit (IMU), RSSI, and IBST tracks in dense deployment. (**b**) The resulting IMU, RSSI, and IBST tracks in sparse deployment. (**c**) Location error of IMU, RSSI, and IBST from actual track in Figure 10a. (**d**) Location error of IMU, RSSI, and IBST from actual track in Figure 10b.

**Figure 11:**
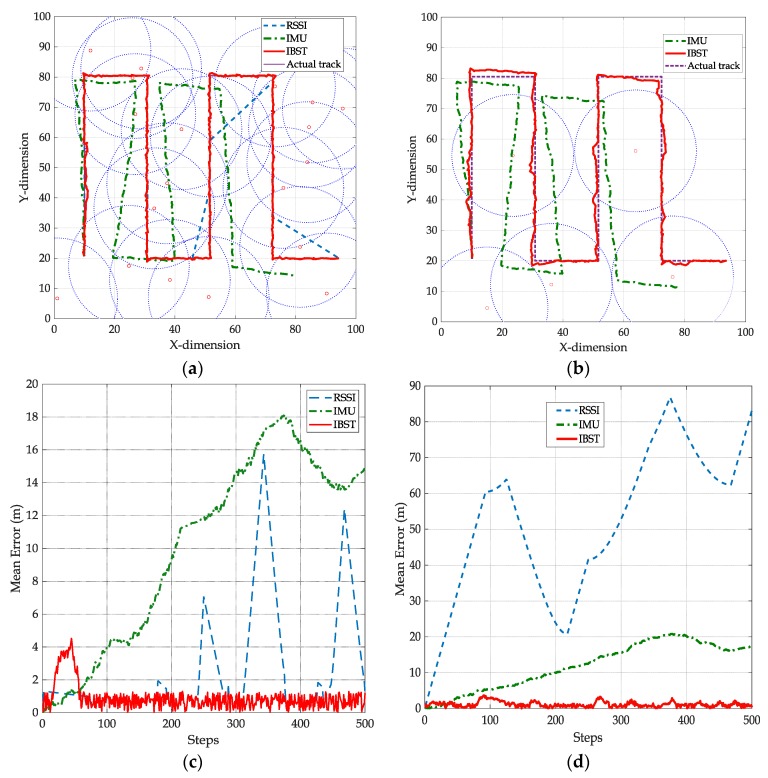
Rectangular back-and-forth track scenario with dense and sparse and random reader deployment (**a**) the resulting IMU, RSSI, and IBST tracks in dense deployment. (**b**) The resulting IMU, RSSI, and IBST tracks in sparse deployment. (**c**) Location error of IMU, RSSI, and IBST from actual track in Figure 11a. (**d**) location error of IMU, RSSI, and IBST from actual track in Figure 11b.

The rectangular back-and-forth track starts at the point (10,20) meters and the circle track starts at (50,20) with respect to a relative Cartesian origin at the bottom left corner of the track area as in Figure 10a,b and Figure 11a,b. The starting point is assumed to be stored in the tag memory; hence, it will be known to the readers once the tag is detected. The values of $\sigma_{a}$ and $\sigma_{v}$ in the covariance matrix $Q_{k}$ of the inertial sensors readings process noise are set to 0.1 m/s2 and 0.1 m/s [19]. The log-normal shadowing path loss model is used as the signal propagation model and the value of the noise variance in RSSI ($\sigma_{RSSI}$) is set to two meters with path loss exponent of 3. The selection of the above values is based on the typical settings of low cost IMUs under indoor/outdoor walking speed conditions [19]. As the main focus of this paper is to provide an estimation of the absolute tag location based on inertial sensors readings and overlapped and non-overlapped readers, providing more accurate RSSI measurements or precise inertial sensors readings is beyond the scope of this work. 

### 5.2. Simulation Results

The first scenario is the circular track of the tag with 500 steps to return to the starting x-y coordinate of (50,20). We consider a dense reader deployment of 20 readers in the track area. The readers are deployed randomly, and the tag advertises itself continuously to surrounding readers; if the tag is detected by three or more readers, RSSI location can be estimated; otherwise, RSSI trilateration outage is considered (i.e., location cannot be estimated). The tag in IBST will also advertise itself continuously to the readers, however, as in Algorithm II, the detection by one or more readers will be enough to provide a location estimation. 

Figure 10a shows a scenario of dense and random deployment of 20 readers within a 100 m^2^ localization area. The actual circular track (in purple) and the tracks based on IMU, RSSI, and IBST after 500 steps. Note that the drifting in the IMU readings is significant after two turns, and with no referencing mechanism, the drift from the actual location will continue to accumulate. RSSI is following the actual track whenever the track passes through three readers simultaneously. If an RSSI localization outage occurs, once the tag passes by three or more readers again, the line connecting between the current estimated location and the one before the outage is considered the track during the outage. IBST track, on the other hand, is continuously referenced to the estimated intersection points due to the dense coverage of the readers. 

In Figure 10c, the mean error between the actual track and the track by RSSI, IMU, and IBST is plotted. IMU error is accumulating due to the drift in inertial sensor readings, which causes an incremental, yet cyclic, error as the number of steps increases. RSSI track error is non-cumulative; however, the actual track is not always passing by points where at least three readers’ ranges exist. Note that when RSSI trilateration is not available, a straight line is used to connect the last available and next available locations, causing a deviation from the actual track, which in turn is represented in periodic error areas. IBST error is none-cumulative and not constrained by three readers simultaneous detection; hence, it is the lowest with continuous update to the displacement vector to follow the actual track. The average mean errors for IMU, RSSI, and IBST from the simulation scenario in Figure 10c are 11.47 m, 3.66 m, and 1.43 m, respectively.

Similarly, Figure 10b shows a sparse and random deployment of 5 readers, the tracks from inertial sensors only, an actual track, and IBST. In this deployment scenario, the drifting in the IMU readings is comparable to the one in Figure 10a as IMU readings are not influenced by the number of readers. RSSI, on the other hand, had no estimated locations as the actual track does not pass by three or more readers simultaneously. As a result, no track can be estimated based on RSSI trilateration. Conversely, more deviation is observed in the IBST track from the actual track. This is due to the lower availability of readers, which results in longer inertial sensors readings before finding an intersection point to represent the estimated actual tag location. The effect of lack of any point within the test area that is covered by three readers or more on the performance of IBST is less severe than the RSSI-based track. In fact, the performance of IBST is significantly superior as shown in Figure 10d, with much lower error than both RSSI and IMU. The average mean errors for IMU, RSSI, and IBST from the simulation scenario in Figure 10d are 10.91 m, 32.84 m, and 2.34 m, respectively.

Another example of a localization scenario is depicted in Figure 11a was a dense and random deployment of 20 readers within 100 m^2^ localization area. The track starts at the point (10,20) and is rectangular with sharp turns to emphasis the effectiveness of IBST over IMU. Note that the drifting in the IMU readings is significant after two turns. Between steps 240 and 290, outage in RSSI localization occurs, causing an increased localization error as shown in Figure 11c. The average mean errors for IMU, RSSI, and IBST from the simulation scenario in Figure 11c are 10.25 m, 5.77 m, and 2.40 m, respectively. 

In Figure 11b, a scenario of sparse and random deployment of 20 readers within the localization area. In this scenario, no points on the actual track are covered by three or more readers; hence, RSSI is in an outage for all steps in the track as the estimated location is the start point (10,20). This is reflected in high localization error as shown in Figure 11d. The error of IBST track is higher than the one in Figure 11c as this scenario suffers from the lack of more than three readers to enhance the location estimation. Nevertheless, the mean error is significantly lower than IMU and RSSI. The average mean errors for IMU, RSSI, and IBST from the simulation scenario in Figure 11d are 10.47 m, 51.69 m, and 1.69 m, respectively.

The above circular and rectangular tracks were executed 1000 times each for:

(a) 5, 10, 20 readers
(b) 5, 20 m reader ranges

The mean error in meters (for 1000 runs) in addition to standard deviation of such mean are provided in Table 3 and Table 4, for circular and rectangular tracks, respectively. The superiority of the IBST technique is evident. The number and range of readers affect the accuracy of both RSSI and IBST. However, RSSI is more prone to localization errors in small and large numbers of readers with high variance around the mean error. This is because of the dependency on passing through an area covered by three or more readers. IBST, on the other hand, is less dependent on the number and range of readers. The reason behind this stability of IBST mean error is that passing by a single reader will provide a location that is near the actual track. IMU is independent of the number or range of readers. However, the drifting in IMU readings without referencing causes a consistent high error in both track scenarios.

We would like to stress here that IBST is a location estimation algorithm that combines asynchronous inertial and range readings to estimate the location; IBST is not an optimization algorithm of ranging nor inertial sensor readings. Therefore, any improvement in ranging methods can replace RSSI in this work (e.g., AoA, TDoA, hybrid RSSI-TDoA, etc.). Similarly, any enhancement in inertial sensors accuracy or drift-reduction algorithms can replace the “IMU” results. Note that any reduction in inertial sensor or ranging errors will improve the accuracy of IBST, as well.

## 6. Conclusions

Solutions proposed in the literature for of object localization require a minimum number of simultaneous range information about the object at any given time. These minimum “sufficient” readings are not always available, especially in IoT dynamic settings where providing centralized and fixed infrastructure is infeasible. In this paper, we proposed a novel cooperative tag-localization system called Inertial-Based Shifting and Trilateration (IBST). In IBST, we leverage crowdsourcing to estimate RFID tag locations in both indoor and outdoor environments, even at the absence of simultaneous reader detections to perform trilateration. IBST does not require any processing by the low power tags, maintaining the system scalability. In our proposed system, crowdsourcing fixed or mobile readers detect battery-assisted tags attached to mobile objects, read their current memory, write detection information on these tags, and report the detected tags’ information to a backend server responsible for users’ queries about objects of interest. IBST uses asynchronous detection information and internal inertial sensor information to enhance localization when the concurrent detection information is not sufficient to localize a tag. During any tag detection, IBST maintains into the tag’s memory: temporal and spatial information *w.r.t.* itself, Absolute Tag Location (ATL) identified by time of calculation, and Tag Displacement Vector (TDV) that are measured based on inertial sensors (IS) records. At insufficient spatial information, IBST shifts (expands the radius) asynchronous RSSI measures based on the tag’s IS records and the recent detection and uses those shifted measures, along with the recent detection, to localize this tag. 

We evaluate IBST’s performance through extensive simulations using MATLAB, and our findings show that IBST outperforms other techniques in sparse and random reader deployment scenarios. IBST can consistently estimate location and give precise and stable localization results in the absence of synchronous detection information, typical scenarios in IoT settings. As future work, we plan to account for non-circular antenna radiation patterns at tags and readers and to consider AoA and TDOA ranging at readers. Furthermore, the incorporation of magnetic field sensors (in addition to inertial sensors) to assist in determining displacement vectors orientation is a future direction to enhance localization accuracy.

## Figures and Tables

**Table 1:** Schema of a Detections table.

Field	Description
time	The time at which a Reader *R_m_* detects tag *T_n_* and creates the detection record.
position	The 2D position of the Reader *R_m_* at time of detection is represented by relative x, y coordinates.
distance	The tag to Reader distance, measured using RSSI.

**Table 2:** Schema of an Absolute Tag Location table.

Field	Description
time	The time at which a Reader *R_m_* estimates the location of tag *T_n_* based on the tag’s detection information.
location	The estimated location of *T_n_*, is represented by x, y coordinates.

**Table 3:** Average error (in meters) after 10^3^ runs of circular track.

Reader Range ↓	Readers Number ↓	Location Estimation Method →	RSSI	IMU	IBST
5 m	5	Mean Error	31.9257	11.3720	5.5248
		$\sigma_{error}$	0.0000	1.5261	2.6642
	10	Mean Error	31.9257	11.2789	3.0226
		$\sigma_{error}$	0.0000	1.6527	1.9542
	20	Mean Error	31.8937	11.1036	2.2547
		$\sigma_{error}$	0.2023	1.1713	1.0244
20 m	5	Mean Error	31.3701	10.8083	2.3412
		$\sigma_{error}$	1.4106	1.5968	0.7357
	10	Mean Error	25.2819	11.3305	1.8249
		$\sigma_{error}$	5.7351	1.4866	0.5001
	20	Mean Error	6.4204	11.6785	1.3353
		$\sigma_{error}$	4.2260	2.1599	0.2438

**Table 4:** Average error (in meters) after 10^3^ runs of rectangular track.

Reader Range ↓	Readers Number ↓	Location Estimation Method →	RSSI	IMU	IBST
5 m	5	Mean Error	51.7140	10.2056	6.3736
		$\sigma_{error}$	0.0002	1.5320	2.8419
	10	Mean Error	51.6840	9.6237	4.0300
		$\sigma_{error}$	0.0005	1.0847	1.1185
	20	Mean Error	49.4478	10.2517	3.5279
		$\sigma_{error}$	8.0887	1.5405	1.5634
20 m	5	Mean Error	39.5628	9.5347	2.0329
		$\sigma_{error}$	14.9362	1.6884	0.9439
	10	Mean Error	18.2600	10.0687	1.8215
		$\sigma_{error}$	4.9584	1.4449	0.4552
	20	Mean Error	5.7765	10.1530	1.6174
		$\sigma_{error}$	4.7883	1.4679	0.1680
